# Anti-cancer effects of dopamine in human glioma: involvement of mitochondrial apoptotic and anti-inflammatory pathways

**DOI:** 10.18632/oncotarget.19691

**Published:** 2017-07-29

**Authors:** Yu-Long Lan, Xun Wang, Jin-Shan Xing, Zhen-Long Yu, Jia-Cheng Lou, Xiao-Chi Ma, Bo Zhang

**Affiliations:** ^1^ Department of Neurosurgery, The Second Affiliated Hospital of Dalian Medical University, Dalian 116023, China; ^2^ Department of Neurosurgery, The Third People’s Hospital of Dalian, Non-Directly Affiliated Hospital of Dalian Medical University, Dalian 116033, China; ^3^ Department of Pharmacy, Dalian Medical University, Dalian 116044, China; ^4^ Department of Physiology, Dalian Medical University, Dalian 116044, China

**Keywords:** dopamine, glioma, mitochondrial apoptotic pathway, inflammation, growth

## Abstract

Despite the emergence of innovative cancer treatment strategies, the global burden imposed by malignant glioma is expected to increase; thus, new approaches for treating the disease are urgently required. Dopamine, a monoamine catecholamine neurotransmitter, is currently regarded as an important endogenous regulator of tumor growth. Dopamine may play an important role in glioma treatment; however, the mechanism underlying the anti-tumor activity of dopamine remains poorly understood. Here, we explored the potential roles of dopamine in glioma and highlight the importance of endogenous regulators of tumor growth. We report that dopamine inhibited glioma cell proliferation. We investigated the biological functions of dopamine via migration, colony formation and apoptosis assays in glioma cells. We also evaluated cytochrome c release from the mitochondria and p50 and p65 subcellular localization by fluorescence microscopy. We performed western blotting and real-time quantitative polymerase chain reaction to detect apoptosis and inflammatory marker protein and gene expression levels, respectively. NF-κB p50/p65 nuclear localization was analyzed after U87MG and U251 cells were treated with dopamine. The *in vivo* anti-tumor efficacy of dopamine was also analyzed in xenograft mice. Taken together, our results indicated that dopamine induced apoptosis by activating the cytochrome c and caspase-dependent apoptotic pathway. Moreover, dopamine markedly down-regulated inflammation-related protein expression levels and p50/p65 NF-κB nuclear localization in tumor cells, thereby inhibiting increases in tumor weight and size in xenograft mice. Thus, therapies targeting the mitochondrial apoptotic and anti-inflammatory signaling pathways regulated by dopamine may represent promising treatments for human glioma.

## INTRODUCTION

Studies have linked cancer risks to personality types [1]. Dopamine influences emotion and personality traits; thus, it has been proposed that personality traits and emotions may impact the risk of cancer development or growth by modulating dopamine release [2]. Thus, dopamine may be important with respect to the development of anti-cancer treatments. Dopamine is present at lower concentrations in tumor tissues than in benign tissues, and increasing dopamine levels via dopamine treatments seems to inhibit tumor cell proliferation. In addition, Moreno-Smith et al. confirmed that dopamine reduces chronic stress-mediated angiogenesis and thus attenuates tumor growth [3]. Researchers have not yet detected dopamine levels systematically to evaluate their potential roles in glioma; however, researchers have previously investigated dopamine concentrations in patients with other types of cancers [4, 5].

Gliomas are refractory brain tumors that represent approximately 50% of all primary malignant brain tumors. The overall post-diagnosis survival time for patients with glioma is 15-18 months [6]. Although the relative incidence of glioma is low, its poor prognosis and negative impact on quality of life and cognitive function in affected patients makes its management a challenging task. Therefore, new treatments for the disease are urgently needed.

Mitochondria are considered semi-autonomous organelles in the eukaryotic cellular system and possess circular DNA containing genes encoding critically important proteins [7]. As mitochondria are the primary sites of cellular ATP and ROS production, changes in the their function may have dire consequences with respect to cellular fate; thus, gaining an understanding of the role and regulatory effects of mitochondria in glioma is an important issue [8]. Mitochondria-targeted drugs have been shown to act by targeting mitochondrial transmembrane potential (MTP) and Bcl-2 anti-apoptotic family proteins in the mitochondrial membrane [9]. In addition, the findings of the *in vitro* study by Sun et al. [10] indicated that dopamine may hamper the function of the signaling machinery of NF-κB, a central regulator of the inflammatory process that plays a critical role in inflammation. Specifically, NF-κB regulates the expression of a group of proinflammatory mediators, including cyclooxygenase-2 (COX-2), inducible nitric oxide synthase (iNOS), tumor necrosis factor α (TNF-α), and interleukin 6 (IL-6) [11]. Thus, NF-κB signaling is an optimal target for therapies intended to treat inflammation. In addition, MAPK signaling pathways, such as those mediated by p38, JNK, and ERK, are important for NF-κB transactivation or translocation [12]. Therefore, NF-κB nuclear translocation is an active inflammatory response, which suggests that drugs designed to manipulate the process may be useful anti-inflammatory agents [13]. The aim of the current study was to confirm the anti-inflammatory effects of dopamine and determine the role of NF-κB and its upstream regulators in these effects to evaluate the potential of dopamine as an alternative drug treatment for glioma.

In the study by Qin et al. [14], dopamine was shown to inhibit growth and induce vascular normalization in cancer tissues by modulating macrophages. This study showed that dopamine displayed anti-tumor activity in a rat C6 glioma model and thus provided strong evidence indicating that dopamine has potential as a novel therapy for human malignant glioma but currently cannot be used as such because of its toxicity [15]. However, as dopamine is a well-characterized drug whose toxicity is manageable, the results of this study may serve as a basis for the development of pharmacokinetic studies and clinical trials designed to evaluate the efficacy of dopamine as a treatment for glioma. Here, we explored the potential roles of dopamine in glioma to add to the growing literature regarding this topic, highlight the importance of endogenous regulators of tumor growth, and promote the development of new therapeutic approaches for the treatment of malignant cancer.

## RESULTS

### Dopamine inhibited U87MG and U251 cell proliferation and altered cell morphology

**Figure 1:**
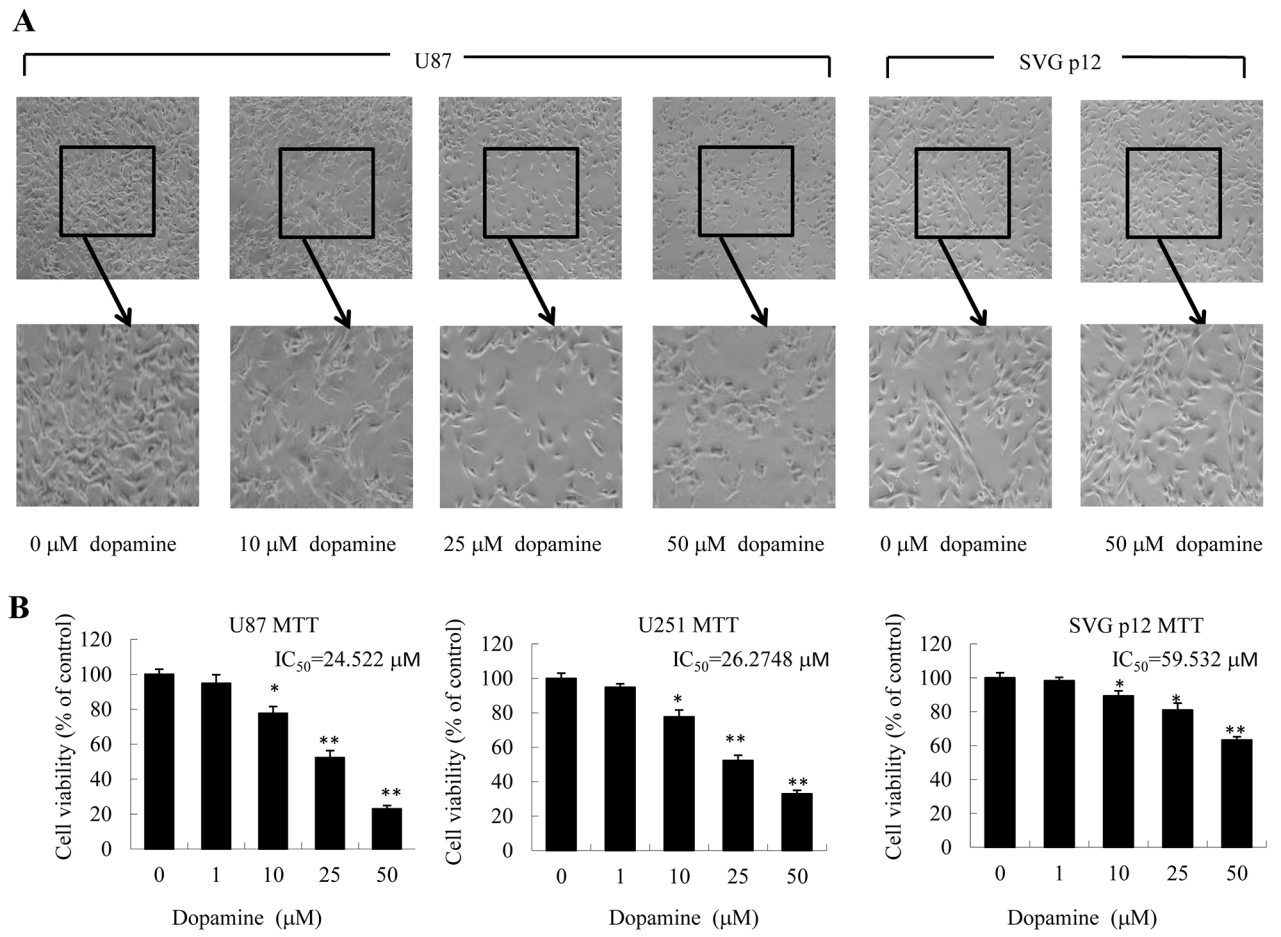
Dopamine inhibited cell viability and altered cell morphology **(A, B)** Human glioblastoma U87MG cells, U251 cells and normal human astrocytes (SVG p12) were treated with dopamine in normal culture medium at the indicated doses. (A) The changes in cell morphology and proliferation in U87MG cells and normal human astrocytes treated with dopamine for 48 h were observed, and the cells were photographed using a microscope fitted with digital camera. (B) At 48 hours after treatment, cell viability was determined by MTT assay. The data are presented as the mean ± SD of three tests. (*P < 0.05, **P < 0.01, significant differences between the dopamine treatment and DMSO vehicle control groups).

First, we quantitatively analyzed the effects of dopamine on U87MG and U251 cell morphology and proliferation by MTT assay. As shown in Figure 1A, dopamine markedly reduced cell-to-cell contact in treated cells compared with control cells, and dopamine-treated cells displayed less proliferation and fewer filopodia than DMSO vehicle control-treated cells. Interestingly, treatment with dopamine at the indicated dose resulted in dose-dependent U87MG and U251 cell growth inhibition but had little effect on normal human astrocyte growth (SVG p12) (Figure 1B).

### Dopamine suppressed glioma cell colony formation, migration and invasion

**Figure 2:**
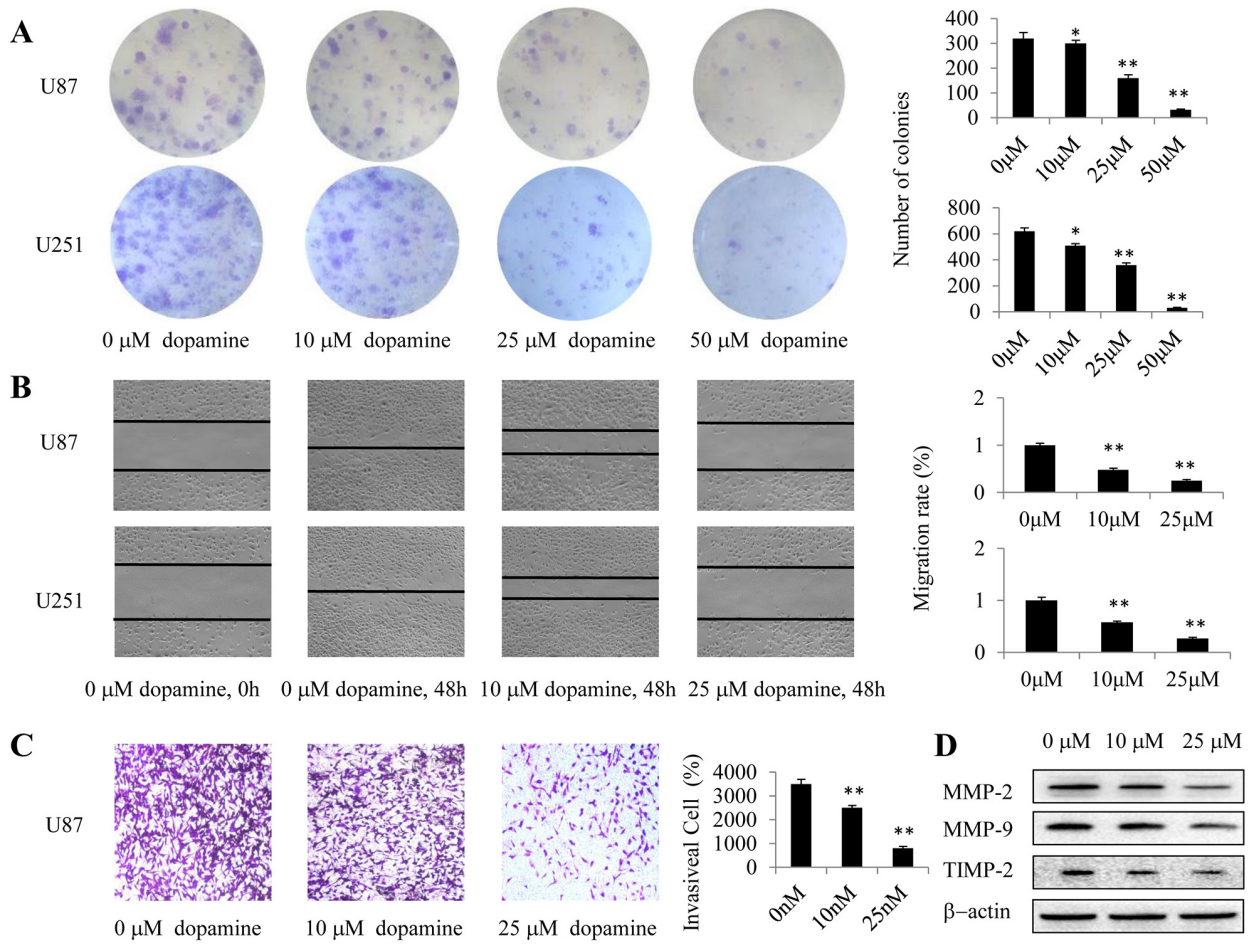
Dopamine suppressed cell colony formation, migration and invasion **(A-D)** Human U87MG or U251 cells were treated with dopamine at the indicated doses for the appropriate time. (A) U87MG- and U251 cell-induced colony formation was analyzed, and the colony formation rates were calculated. (B) Cell migration was also analyzed by wound-healing assay. U87MG and U251 cells were seeded in 6-well plates and grown to full confluence. Cell migration was measured, and the migration rate was calculated. (C) Dopamine inhibited glioma invasion in transwell assay. U87MG cells were plated in transwell chambers pre-coated with Matrigel. The cells that migrated to the bottom of the membrane were counted using an inverted microscope. Original magnification, 40× C. (D) The expression levels of the key proteins related to cell invasiveness were determined by western blotting. (*P < 0.05, **P < 0.01, significant differences between the dopamine treatment and DMSO vehicle control groups).

We employed clonogenic cell survival assay to evaluate the influence of dopamine on U87MG and U251 cell clonogenic capacity. We found that dopamine significantly inhibited colony formation and induced significant decreases in the colony formation ratio (Figure 2A). Wound-healing assay further revealed that dopamine inhibited U87MG and U251 cell mobility (Figure 2B), and quantitative analysis revealed that the effects of dopamine on cell migration were dose-dependent. These results suggest that dopamine is well-suited to suppress U87MG and U251 cell colony formation and migration. In addition, we investigated the effects of dopamine on cell invasion by transwell assays and confirmed that dopamine inhibited U87MG cell invasion (Figure 2C). Moreover, we performed western blotting analysis to assess the expression levels of key proteins related to glioma cell migration and invasion (MMP-2, MMP-9, and TIMP-2) in U87MG cells and confirmed the results of the above experiments (Figure 2D). Taken together, these results showed that dopamine significantly inhibited glioma cell colony formation, migration and invasion in a dose-dependent manner.

### Dopamine treatment increased apoptosis by inducing mitochondrial dysfunction

**Figure 3:**
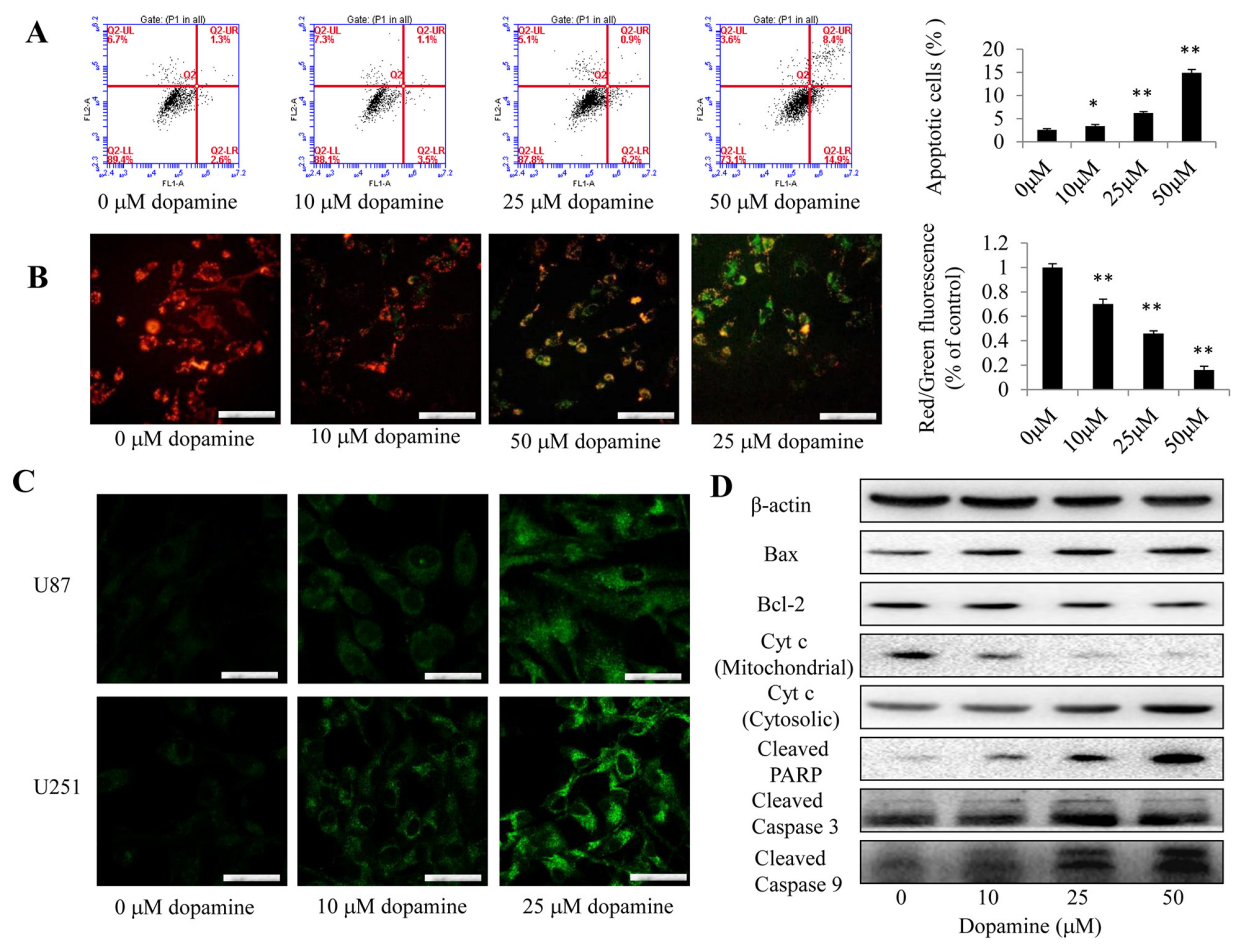
Dopamine treatment induced mitochondrial dysfunction **(A)** Cell apoptosis was assessed by FACS analysis after the indicated cells were treated with dopamine for 24 h. The data are presented as the mean ± SD of three separate experiments. (B) U87MG cells were exposed to dopamine (indicated doses for 24 h), and the mitochondrial membrane potential was determined by JC-1 staining and fluorescence microscopy. Bar = 100 μm. n = 3. **(B)** Quantitative analysis of the shift in mitochondrial fluorescence from orange-red to green in the groups (red/green fluorescence ratio) was conducted. All values are denoted as the mean ± SD from ten independent photographs taken in each group. **(C)** The level of cytochrome c release from the mitochondria in U87MG and U251 cells was determined by fluorescence microscopy. Bar = 50 μm. n = 3. **(D)** The levels of the Bax, Bcl-2, cleaved caspase-3/9, cleaved PARP, and mitochondrial and cytosolic cytochrome c protein in U87MG cells were analyzed by western blotting (significant differences are indicated as **P < 0.01 compared with control cells cultured in complete medium).

Apoptosis was assessed by FACS analysis after the indicated cells were treated with dopamine for 24 h (Figure 3A). Induction of target cell apoptosis is a key mechanism through which anti-cancer therapeutic agents exert their effects [16], and the enhancements of cell growth inhibition induced by dopamine have been shown to be associated with increases in glioma cell apoptosis. The results of the analysis showed that dopamine treatment significantly and dose-dependently induced apoptosis in U87MG cells. In addition, as mitochondria play critical roles in apoptosis, the present study aimed to determine the effects of dopamine on mitochondrial function. JC-1 was used to evaluate mitochondrial membrane potential. Treatment with 10, 25 and 50 μM dopamine for 24 h led to a decrease in mitochondrial membrane potential, a change that manifested as increases in JC-1 green staining intensity and reductions in JC-1 red staining intensity (Figure 3B). Then, the red-to-green fluorescence intensity ratio was determined using a fluorescence microplate reader. The results indicated that dopamine induced a dose- and time-dependent decrease in the ratio (Figure 3B). These results suggest that dopamine triggers mitochondrial dysfunction in U87MG cells. Additionally, some studies have noted that the release of cytochrome c from the mitochondria to the cytosol is a critical step in apoptosis activation [17]. Many stimuli induce cytochrome c release from the mitochondrial intermembrane space into the cytosol, thereby triggering apoptosis. We subsequently monitored the changes in cytochrome c subcellular localization in U87MG and U251 cells by IF to determine whether dopamine can induce cytochrome c release. As shown in Figure 3C, treatment with dopamine (5 μM, 10 μM or 50 nM) significantly triggered the release of cytochrome c from the mitochondria to the cytosol. These results indicated that dopamine induced U87MG and U251 cell apoptosis by triggering cytochrome c release from the mitochondria and facilitating downstream apoptosome assembly and caspase activation in the cytosol.

We next examined the expression of key proapoptotic proteins (Bax, Bcl-2, PARP, caspase-3, caspase-9) in U87MG cells by western blotting. We found that dopamine increased Bax expression, decreased Bcl-2 expression and increased cleaved caspase-3, caspase-9 and PARP protein expression levels in the corresponding group compared with the control group (Figure 3D). In addition, dopamine significantly increased the expression levels of cytochrome c in the cytosolic fraction by reducing the expression of cytochrome c in the mitochondria, suggesting that dopamine-induced cytochrome c release from the mitochondria to the cytosol is a dose-dependent process (Figure 3D).

### Dopamine decreased the expression levels of proinflammatory mediators

**Figure 4:**
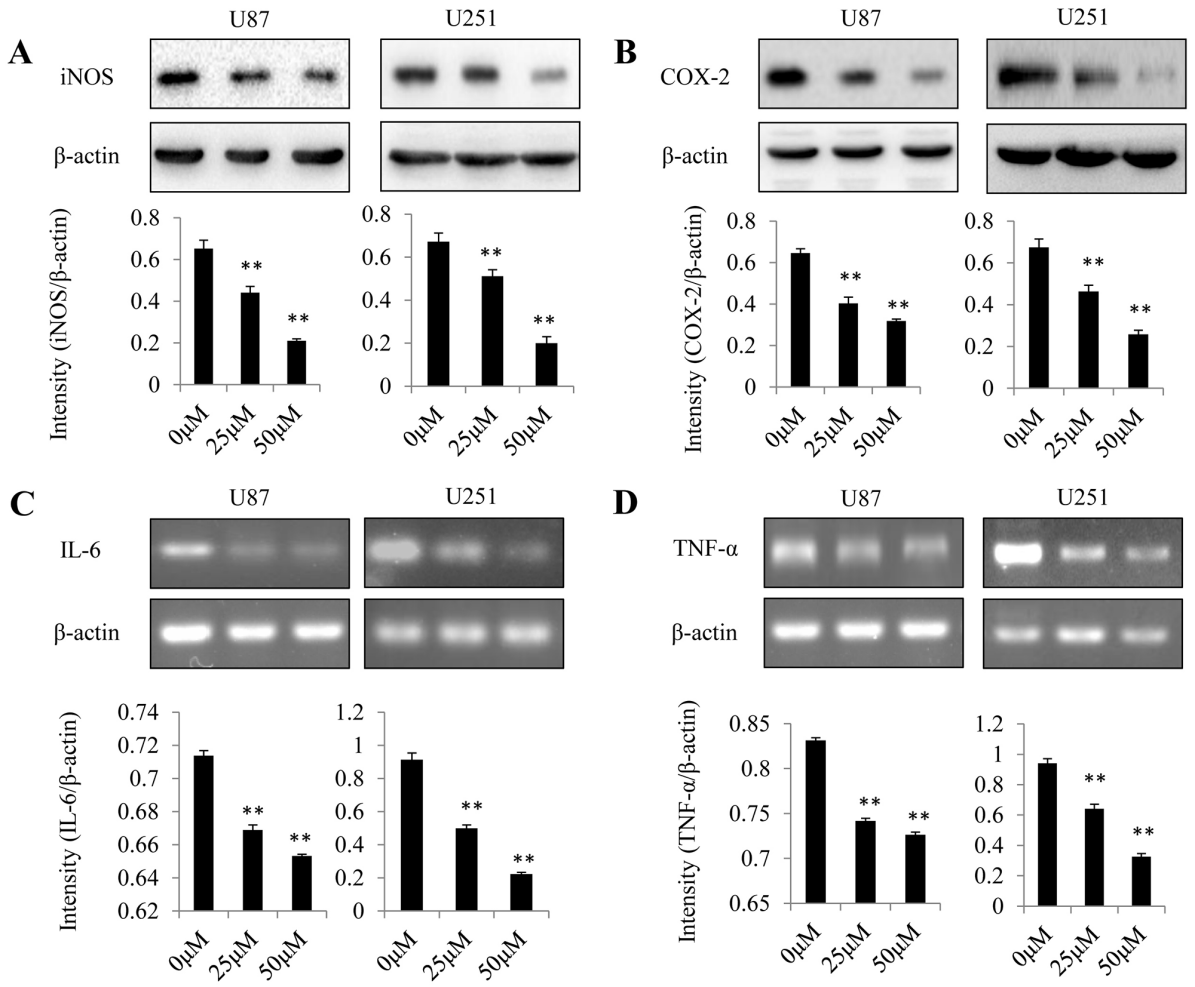
Effect of dopamine on proinflammatory mediator expression in glioma cells Human U87MG and U251 cells were treated with dopamine at the indicated doses. At 48 h after treatment, iNOS and COX-2 protein expression levels were analyzed by western blotting **(A-B)**, and IL-6 and TNF-α gene expression levels were analyzed by RT-qPCR **(C-D)** in U87MG and U251 cells. The data are presented as the mean ± SD of three tests. (**P < 0.01, significant differences between the dopamine treatment and DMSO vehicle control groups).

The expression levels of proinflammatory mediators in U87MG cells were examined to assess the anti-inflammatory effects of dopamine. At 48 h after treatment, we analyzed iNOS and COX-2 protein expression levels by western blotting (Figure 4A and 4B) and IL-6 and TNF-α gene expression levels by RT-qPCR (Figure 4C and 4D). We found that dopamine reduced iNOS and COX-2 protein expression levels and decreased IL-6 and TNF-α gene expression levels in a dose-dependent manner. These results confirm that significant reductions in proinflammatory mediator expression levels were observed in the groups treated with either 25 or 50 μM dopamine compared with those treated with vehicle (P < 0.01). These initial results indicate that dopamine has strong anti-inflammatory effects in glioma cells *in vitro*.

### Dopamine inhibited NF-κB translocation from the cytoplasm to the nucleus

**Figure 5:**
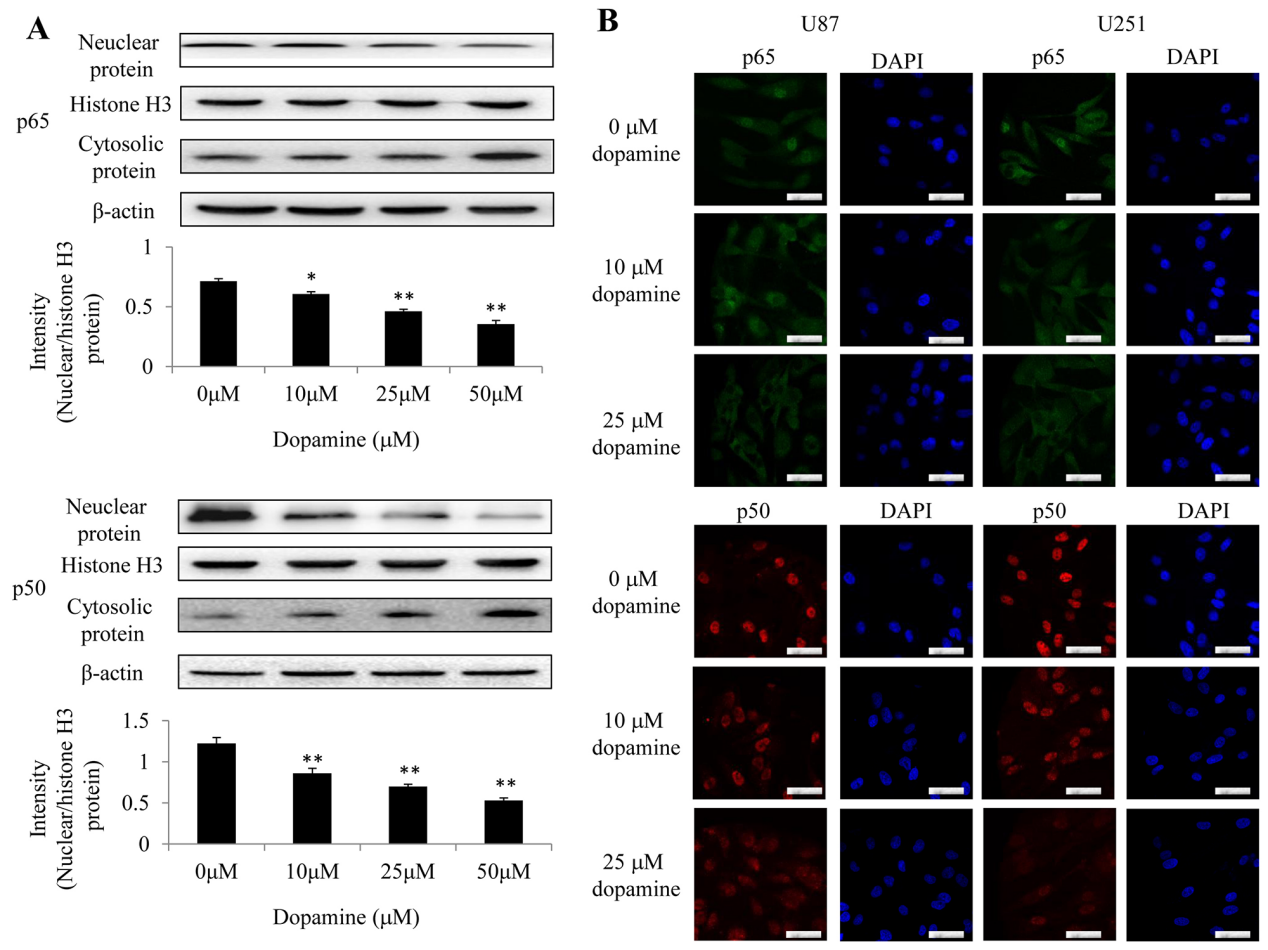
Dopamine inhibited NF-κB translocation from the cytoplasm to the nucleus in glioma cells **(A)** Nuclear and cytosolic NF-κB expression levels were analyzed by western blotting, and a quantitative analysis was performed to compare p65 and p50 translocation levels between the different treatment groups. **(B)** At 24 h after treatment, p50 and p65 subcellular localization was examined by confocal microscopy. More than 100 cells were inspected per experiment, and cells with typical morphology are shown. Bar = 50 μm. n = 3. (*P < 0.05 and **P < 0.01 compared with the control cultures).

Previous reports suggest that NF-κB is an important transcription factor that regulates the expression of inflammatory cytokines and proinflammatory mediators [18]. We detected the constitutive translocation of NF-κB p50/p65 to the cell nucleus in U87MG cells by western blotting (Figure 5A). We next performed IF to confirm the nuclear localization of p50 and p65 in U87MG and U251 cells by confocal microscopy (Figure 5B). We found that treatment with dopamine significantly inhibited the translocation of NF-κB p65/p50 proteins from the cell cytoplasm to the nucleus in the corresponding group compared with the DMSO control group (Figure 5A and 5B). The results indicate that dopamine-induced U87MG and U251 cell proliferation inhibition may be mediated via the inhibition of NF-κB translocation from the cytoplasm to the nucleus.

### Dopamine suppressed p38 and JNK MAPK phosphorylation

**Figure 6:**
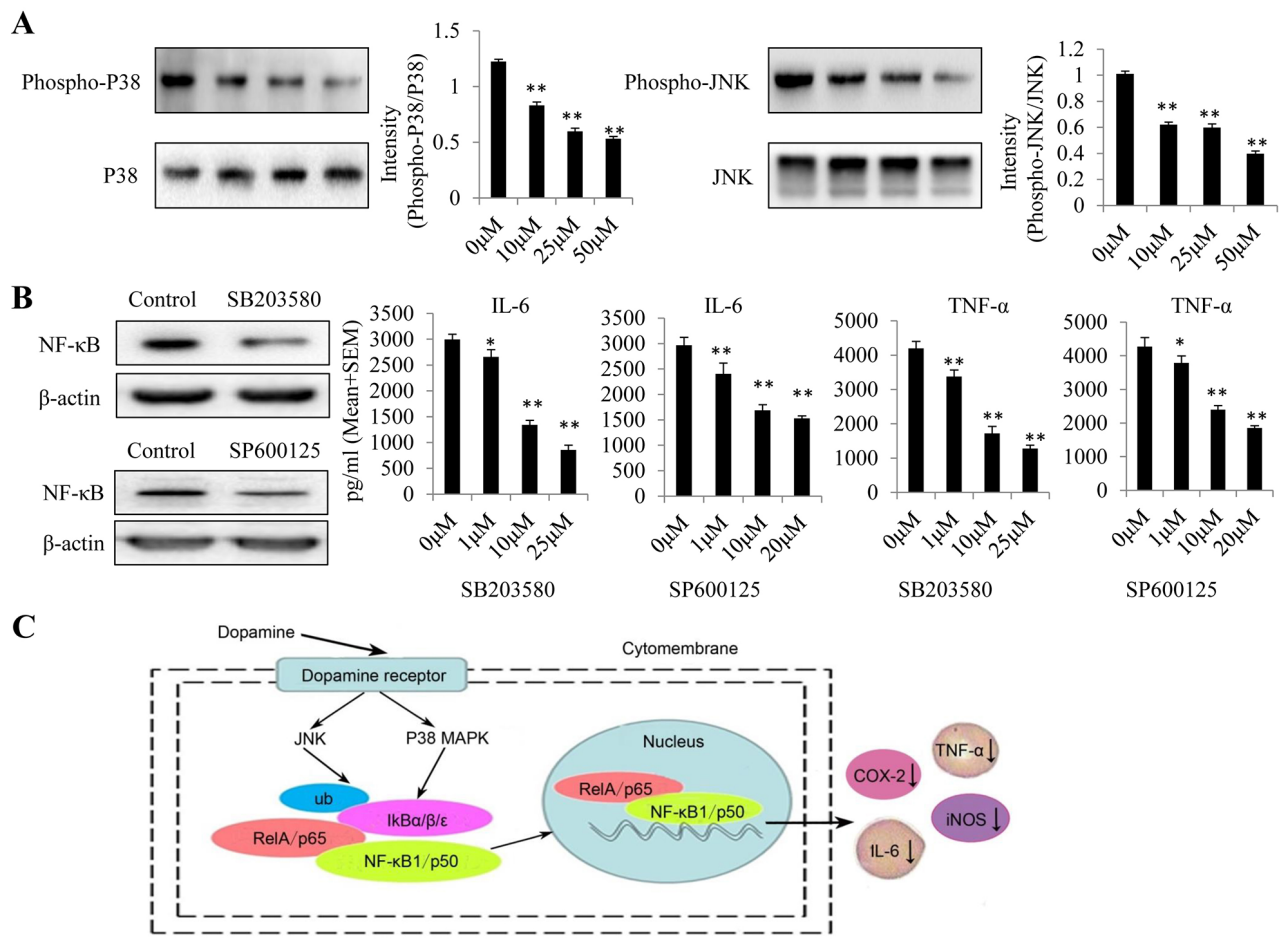
Dopamine suppressed the inflammatory response by partially regulating MAPK signaling by targeting p-p38 and p-JNK in glioma **(A)** Dopamine suppressed P38 and JNK MAPK phosphorylation in U87MG cells. **(B)** Effects of P38 and JNK inhibitors (SB203580 and SP600125) on the suppression of NF-κB expression and TNF-α and IL-6 production in glioma cells. The cells were incubated with the P38 and JNK inhibitors at the indicated concentrations for 24 h. After the cells had incubated for 24 h, the cell lysates were collected to test NF-κB expression levels in each group. In addition, the supernatant was collected, and proinflammatory cytokine levels were measured by ELISA. *P < 0.01 and **P < 0.01 compared with the control cultures. n = 3. **(C)** The anti-inflammatory mechanism through which dopamine exerts its effects on the NF-κB signaling pathway.

MAPK p38 and JNK isoforms are the best-studied members of the MAPK family in mammalian cells [19]. As inflammation induces hyper-phosphorylation and activation of several members of the MAPK family, namely, JNK, p38 and ERK [20], we investigated the phosphorylation status of these MAPKs by western blotting analysis (Figure 6A). We found that dopamine suppressed p38 and JNK MAPK phosphorylation in U87MG cells but did not significantly affect ERK MAPK phosphorylation (data not shown). In addition, we used the indicated p38 and JNK inhibitors (SB203580 and SP600125) to examine the role of MAPK signaling in the anti-inflammatory effects of dopamine. We found that the p38 and JNK inhibitors inhibited NF-κB activation and IL-6/TNF-α production in a dose-dependent manner (Figure 6B). All of these results indicate that dopamine suppresses the inflammatory response by partially regulating MAPK signaling by targeting p-p38 and p-JNK in glioma cells. The anti-inflammatory mechanisms through which dopamine exerts its effects on the NF-κB signaling pathway are displayed in a graphical representation (Figure 6C). The molecular effects of dopamine will be the subject of our next investigation.

### Dopamine inhibited the growth of glioma xenografts in nude mice

**Figure 7:**
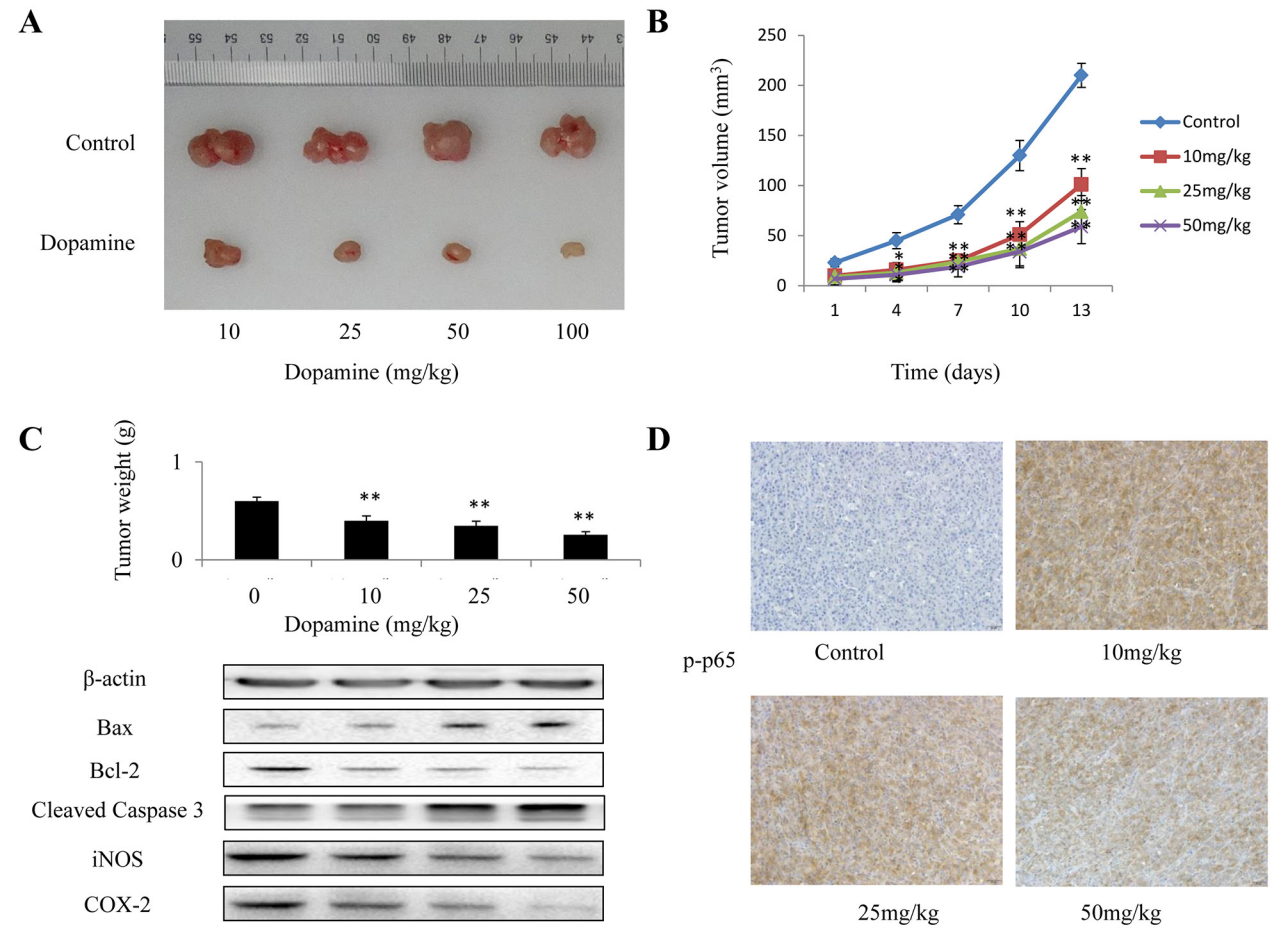
The *in vivo* anti-tumor efficacy of dopamine was analyzed in xenograft mice In the U87MG glioma subcutaneous model, 5 × 10^6^ human U87MG cells were subcutaneously injected into the right flanks of the mice. When the tumors reached a size of approximately 100 mm^3^, the mice received dopamine (0, 10, 25, 50 and 100 mg/kg) or vehicle intraperitoneally once every three days for 13 days. **(A-C)** The mice were euthanized at 24 h after the completion of treatment, and the tumor volumes and weights were measured. In addition, the expression of some apoptosis and inflammatory markers, such as caspase-3, Bax, Bcl-2, COX-2 and iNOS, were measured. **(D)** Immunohistochemical analysis of p-p65 protein expression in the tumor samples. Neutral formalin-fixed tumor samples were prepared from the animals and analyzed by immunohistochemical staining with rabbit anti-rabbit secondary antibodies using a Vectastain Elite ABC Kit and examined under a microscope. The values are presented as the mean ± SD, n = 3. (**P < 0.01 compared with the control cultures).

We explored the potential of dopamine as a novel molecular therapeutic agent in mice with human glioma xenografts. Mice bearing subcutaneous tumors were treated with dopamine for 13 days after tumor cell injection. The mice were divided into three treatment groups (Figure 7A). Tumor volume (Figure 7B) and tumor weight (Figure 7C) in dopamine-treated mice decreased significantly compared with those in control mice post-treatment. In addition, the expression levels of some apoptosis and inflammatory markers, namely, caspase-3, Bax, Bcl-2, COX-2 and iNOS, were measured and were found to have decreased in dopamine-treated mice compared with control mice (Figure 7C). Moreover, immunohistochemical staining assay was used to determine the expression of p-p65, the activated form of p65. p-p65 expression levels were significantly decreased in the group treated with dopamine *in vivo* compared with the group treated with vehicle (Figure 7D). These results indicated that dopamine inhibited human glioma cell growth in the xenograft.

## DISCUSSION

Dopamine is an important catecholamine neurotrans-mitter that exerts its effects in its target organs by binding to dopamine receptors (DRs), namely, DR1 and DR2 [21, 22]. *In vitro* studies have shown that dopamine inhibits tumor cell proliferation in ovarian cancer and Hodgkin’s lymphoma [23, 24]. However, it does not affect colon and breast cancer cell proliferation [25, 26]. Currently, the precise mechanisms underlying the inhibitory effects of dopamine on cancer development are poorly understood. We accomplished the following in the present study: first, we provided direct evidence indicating that administering dopamine at nontoxic doses inhibits tumor growth in human U87MG and U251 glioma cells. To our knowledge, this is the first study to directly demonstrate that the dopamine can inhibit human glioma growth both *in vitro* and *in vivo*. Second, we demonstrated for the first time that dopamine retards tumor growth through mitochondrial apoptotic and anti-inflammatory pathways.

In the present study, we found that dopamine could effectively inhibit glioma cell growth and enhance apoptosis in a dose-dependent manner. The IC_50_ of dopamine was only 25 μM; however, dopamine had little effect on normal human brain cells at a higher dosage. Furthermore, we showed that the effects of dopamine on brain cancer cell growth and apoptosis were mediated through cytochrome c/caspase-dependent apoptotic and anti-inflammatory pathways. Dopamine inhibited the translocation of the NF-κB p65/p50 proteins, which exert key effects in the pathogenesis of inflammation, from the cell cytoplasm to the nucleus. To the best of our knowledge, this study may be the first to report the effects of treatment with dopamine on the expression profiles of NF-κB subunits in glioma and to demonstrate the mechanisms underlying the anti-cancer effects of dopamine both *in vitro* and *in vivo*.

Given their essential roles in energy metabolism and the regulation of apoptosis-related signaling cascades, mitochondria are considered attractive targets for cancer therapies. Several agents impacting mitochondrial function have been found to exert anti-cancer effects in both *in vitro* and *in vivo* models, and one common feature displayed by these compounds is their ability to preferentially kill cancer cells while inducing low cytotoxicity in normal cells [27]. In the present study, mitochondrial membrane potential was found to be altered by treatment of dopamine. Furthermore, the release of cytochrome c from the mitochondria to the cytosol resulted in final apoptosis activation. Intriguingly, a high Bax/Bcl-2 ratio is the most important indicator of apoptosis mediated through the intrinsic mitochondrial mechanism [28], and dopamine induced increases in the Bax/Bcl-2 ratio, cytochrome c release and caspase protein activation (Figure 3). Thus, the data from the present study indicated that dopamine treatment induced mitochondrial apoptotic pathway activation.

One of the pivotal factors in the inflammatory processes is NF-κB, which can rapidly induce the production of inflammatory mediators, such as COX-2, iNOS, TNF-α and IL-6 [11]. We observed that dopamine can inhibit the expression of inflammatory mediators. The intracellular pathways that are involved in inflammatory reactions are complicated; thus, herein, we investigated the precise mechanisms by which dopamine inhibits its cellular targets. We also sought to explore its effects on NF-κB activity and found that it could suppress NF-κB nuclear translocation. MAPK pathways play pivotal roles in regulating inflammation and inflammatory mediator production, and the results of the present study showed that dopamine specifically targets p-p38 and p-JNK, which is a very interesting finding. Studies assessing the molecular mechanisms by which the small structural identity of dopamine induces specific recognition and inhibition of target MAPKs are underway. In conclusion, as illustrated above, dopamine suppressed inflammation in glioma cells by suppressing NF-κB and p-p38 and p-JNK MAPKs. Applying previously used drugs has great promise with respect to the treatment of various diseases, especially when the application of specific drugs is substantiated by mechanistic reasoning. Our results also suggest that dopamine may be an endogenous bioactive compound with anti-inflammatory properties and may thus be a useful agent for treating malignant gliomas and various other inflammation-mediated pathological conditions. However, hypoxia is considered an important cause of therapeutic resistance, and anti-cancer therapeutic regimens comprising single agents are relatively ineffective with respect to treating glioma. Thus, it is very important to determine the efficacy of drugs under both normoxia and hypoxia, which may be a direction for future studies.

## MATERIALS AND METHODS

### Antibodies and other materials

The primary antibodies to COX-2, iNOS, IL-6, TNF-α, p38, JNK, ERK, p-p38, p-JNK, p-ERK, Bax, Bcl-2, cleaved caspase-3, cleaved caspase-9, cleaved PARP, NF-κB, NF-κB p65 and p-p65, β-actin, and histone H3, as well as all the corresponding secondary antibodies, were obtained from Cell Signaling Technology (Cell Signaling Technology, Inc, USA), and the primary antibodies to NF-κB p50 and cytochrome c were obtained from Santa Cruz Biotechnology (Santa Cruz, CA, USA). Trypsin, Dulbecco’s modified Eagle’ medium (DMEM) and fetal bovine serum (FBS) were obtained from HyClone Laboratories (HyClone Laboratories Inc.), and dopamine (DA; H8502) was purchased from Sigma-Aldrich. For the experiments, we prepared a solution of dopamine (10 mM) in distilled water, which served as a stock solution and was stored at -20°C. Dopamine, which was stable in distilled water, was diluted in culture medium to the concentration at which it was used in the experiments. Phosphate buffered saline (PBS), protease inhibitor cocktail and 5-diphenyltetrazolium bromide (MTT) were purchased from Sigma Chemical Co (St. Louis, MO), and the all other chemicals used herein were purchased from Sigma Chemical Co. (St. Louis, MO), unless otherwise specified.

### Cell culture

The human glioma cell lines were obtained from ATCC (Manassas, VA). The cells were maintained in DMEM supplemented with 10% FBS. All the cell cultures were maintained at 37°C in a humidified atmosphere containing 5% CO2.

### Cell viability assay

Cell viability was determined by MTT assay (Roche Diagnosis, Indianapolis, IN). Briefly, the cells were seeded in 96-well plates at a density of 6 × 10^3^ cells/well. The cells were allowed to adhere overnight before being placed in fresh medium containing various concentrations (0, 10, 25, 50, 80 μM) of dopamine dissolved in DMSO (final concentration, 0.1%). After the cells had incubated for 48 h, we measured their growth. We quantified the effects of dopamine on cell viability by comparing the percentage of viable cells in the treated group with that in the untreated control group. The cell viability percentage of the control group was arbitrarily assigned a value of 100%. The dopamine concentration required to cause 50% cell growth inhibition (IC_50_) was determined through analyses of dose-response curves. The OD values were determined. All experiments were performed in triplicate.

### *In vitro* migration assay

Scratch assay (wound healing assay) was performed to assess cell migration. The abovementioned cells were grown to full confluence in six-well plates, wounded with a sterile 100-μl pipette tip after 6 h of serum starvation and then washed with starvation medium to remove detached cells from the plates. The cells were then treated with the indicated doses of dopamine in full medium and stored in a CO_2_ incubator. After 48 h, the medium was replaced with PBS, the wound gap was observed, and the cells were photographed with a Leica DM 14000B microscope fitted with digital camera.

### Colony formation assay

To analyze the sensitivity of the cells to dopamine, we performed an *in vitro* colony formation assay. Briefly, U87MG and U251 cells (0.8 × 10^3^ per well) were seeded in six-well plates containing 2 ml of growth medium with 10% FBS and cultured for 24 h. Then, the medium was removed, and the cells were exposed to various concentrations of dopamine (0, 10, 25 and 50 μM). After 24 h, the cells were washed with PBS and suspended in fresh medium containing 10% FBS. The cultures were maintained at 37°C in a 5% CO2 incubator for 14 days, which allowed the viable cells to grow into macroscopic colonies. Then, the medium was removed, and the colonies were counted after being stained with 0.1% crystal violet.

### Transwell invasion assay

The motility of U87MG was performed in 24-well transwell plates. The upper surface of polycarbonate filters with 8 μm pores was coated with 75 μL Matrigel (Matrigel: DMEM=1:3) and incubated for 0.5 h at 37°C for gelling. Then, Cells were seeded into the upper chambers at a density of 5 × 10^4^ cells per chamber, the bottom chamber were filled with 600 μL DMEM with 10% FBS. Both top and bottom chamber contained the same concentrations of dopamine. After 24 h incubation, non-invasive cells on the upper membrane surfaces were removed by wiping with cotton swabs. Invaded cells were fixed with methanol and stained with 0.1% Crystal Violet Staining Solution. The membrane was dried in the air. Images were taken using a Leica DM 14000B microscope. Cell invasion counted in five independent areas per membrane. The results were the means calculated from five replicates of each experiment.

### Apoptosis assay

Apoptosis was measured by a fluorescence-activated cell sorter (FACS) using an Annexin V-FITC Apoptosis Detection Kit (Nanjing KeyGEN Biotech. CO., LTD.). Briefly, the cells were plated in 6-well plates and then treated with dopamine. After 12 h of treatment, the cells were collected and washed once with cold PBS before being stained simultaneously with FITC-labeled annexin V and PI and then analyzed using an FACS Accuri C6 (Genetimes Technology Inc.).

### Mitochondrial membrane potentials assay

A JC-1 probe was employed to measure mitochondrial depolarization in glioma cells. Briefly, the cells were cultured in six-well plates after receiving the indicated treatments and then incubated with equal volumes of JC-1 staining solution (5 μg/ml) for 20 min at 37°C before being rinsed twice with PBS. Mitochondrial membrane potential was monitored by measuring the relative amounts of dual emissions from mitochondrial JC-1 monomers or aggregates under Argon-ion 488-nm laser excitation using an Olympus fluorescence microscope. Mitochondrial depolarization was indicated by decreases in the red/green fluorescence intensity ratio.

### Western blotting analysis

The cell lysate proteins were separated by electrophoresis on a 7.5-12% sodium dodecyl sulfate-polyacrylamide minigel (SDS-PAGE) and then electrophoretically transferred to a PVDF membrane. The blots were probed with the appropriate specific antibodies, and the protein bands were detected by enhanced chemiluminescence. Similar experiments were performed at least three times. The total protein concentration was determined using a BCA Protein Assay Kit.

### Quantitative real-time reverse transcription polymerase chain reaction (RT-qPCR)

Total RNA was extracted from the glioma cells after they had been treated with dopamine for 48 h using TRIzol reagent, according to the kit protocol (TaKaRa Bio, Dalian, China). cDNA was reverse-transcribed using a PrimeScript RT Reagent Kit (TaKaRa Bio, Dalian, China), according to the manufacturer’s instructions. The Q-PCR reaction was performed according to the kit protocol (TaKaRa Bio, Dalian, China), and amplification was performed using a Mx3005P Real-Time PCR System (Agilent, CA, USA). The relative mRNA expression levels of each gene were normalized to the RNA expression levels of GAPDH and analyzed using the 2^−ΔΔCT^ method. The primers were synthesized by Invitrogen (Shanghai, China).

### Enzyme-linked immunosorbent assays (ELISA)

The cells were incubated with dopamine at the indicated concentrations for 24 h, after which the supernatant was collected, and proinflammatory cytokine levels were measured by ELISA. IL-6/TNF-α levels in the culture medium and cells were determined with an IL-6/TNF-α Emax Immuno Assay System, according to the manufacturer’s instructions. A standard reference curve was prepared for each assay to accurately quantify the levels of the above proinflammatory cytokines in the experimental samples.

### Confocal immunofluorescence

IF staining was performed in cells cultured in chamber slides. After dopamine treatment, the cells were washed in PBS and fixed with 4% paraformaldehyde for 10 min at room temperature (RT). The samples were then permeabilized with 0.2% TritonX-100 for 5 min before being blocked with 10% bovine serum albumin (BSA) in PBS for 30 min. Antibodies against cytochrome c, p65 and p50 in 1% blocking solution were subsequently added to the samples, which were incubated overnight at 4°C. Following three 10-min washes with PBS, the cells were treated with fluorescein isothiocyanate- and rhodamine-conjugated secondary antibodies in 1% blocking solution and incubated for 1 hr. Then, the stained samples were mounted with 4’, 6-diamidino-2-phenylindole (DAPI)-containing Vectashield solution (Vector Laboratories Inc.) to counterstain the cell nuclei. After five additional 5-min washes, the samples were examined with a Leica DM 14000B confocal microscope.

### Animal studies

All the animals were maintained in the SPF Laboratory Animal Center at Dalian Medical University, which was also the site at which all the animal experiments were performed. Female nu/nu mice (4-6 weeks old) were used for these experiments. To evaluate the therapeutic efficacy of dopamine in a human U87MG orthotopic glioma mouse model, we subcutaneously injected U87MG cells (2 × 10^6^ in 100 μl PBS) near the axillary fossae of the nude mice using a 27-gauge needle. The tumor cell-inoculated mice were then randomly divided into three treatment groups, each of which contained five mice. Two weeks later, when the tumors had reached a size of 3 mm × 4 mm, group A was treated with PBS, and groups B and C were treated with daily intraperitoneal injections of dopamine. The tumors were measured with a caliper every 3 days, and tumor volumes were calculated using the following formula: V = 1/2 (width2 × length). Body weights were also recorded. On day 30 after tumor cell inoculation, all the experimental mice were euthanized with ether anesthesia, and their total tumor weights were measured. To determine p65 NF-κB expression levels, we harvested the tumor tissues and fixed them with 10% neutral formalin before desiccating them and embedding them in paraffin. Four-micrometer sections were subsequently stained with hematoxylin and eosin and p-p65 NF-κB (1:150) antibodies and examined under a light microscope. The images were obtained a Leica DM 4000B fluorescence microscope equipped with a digital camera.

All animal maintenance and procedures were carried out in strict accordance with the recommendations established by the Animal Care and Ethics Committee of Dalian Medical University as well as the guidelines by the U.S. National Institutes of Health Guide for the Care and Use of Laboratory Animals. The protocol was approved by the Animal Care and Ethics Committee of Dalian Medical University. In animal study, all efforts were made to minimize suffering of mice. All mice were humanely sacrificed by ether anesthesia inhalation before death.

### Statistical analysis

All experiments were repeated three times. Data are represented as mean ± standard deviation (SD). Analysis of variance and Student’s t-test were used to compare the values of the test and control samples *in vitro* and *in vivo*. P < 0.05 was considered to be a statistically significant difference. SPSS 17.0 software was used for all statistical analysis.

## CONCLUSIONS

In conclusion, we found that dopamine increases apoptosis by inducing mitochondrial dysfunction and suppresses inflammation-related signaling pathways in glioma both *in vivo* and *in vitro*. Our mechanistic investigations revealed that dopamine may target NF-κB and, more specifically, p-p38 and p-JNK MAPKs. Dopamine is a well-characterized drug with manageable toxicity, and the results of our study may serve as a basis for the development of pharmacokinetic studies and clinical trials designed to evaluate the anti-cancer effects of dopamine, which may have potential as an effective agent in glioma treatment
